# A low-coverage 3′ RNA-seq to detect homeolog expression in polyploid wheat

**DOI:** 10.1093/nargab/lqad067

**Published:** 2023-07-12

**Authors:** Jianqiang Sun, Moeko Okada, Toshiaki Tameshige, Rie Shimizu-Inatsugi, Reiko Akiyama, Atsushi J Nagano, Jun Sese, Kentaro K Shimizu

**Affiliations:** Research Center for Agricultural Information Technology, National Agriculture and Food Research Organization, 3-1-1 Kannondai, Tsukuba, Ibaraki 305-8517, Japan; Department of Evolutionary Biology and Environmental Studies, University of Zurich, Winterthurerstrasse 190, 8057 Zurich, Switzerland; Kihara Institute for Biological Research, Yokohama City University, 641-12 Maioka, Totsuka-ward, Yokohama, Kanagawa 244-0813, Japan; Kihara Institute for Biological Research, Yokohama City University, 641-12 Maioka, Totsuka-ward, Yokohama, Kanagawa 244-0813, Japan; Division of Biological Sciences, Graduate School of Science and Technology, Nara Institute of Science and Technology, 8916-5, Takayama-cho, Ikoma, Nara 630-0192, Japan; Department of Evolutionary Biology and Environmental Studies, University of Zurich, Winterthurerstrasse 190, 8057 Zurich, Switzerland; Department of Evolutionary Biology and Environmental Studies, University of Zurich, Winterthurerstrasse 190, 8057 Zurich, Switzerland; Faculty of Agriculture, Ryukoku University, Yokotani 1-5, Seta Ohe-cho, Otsu, Shiga 520-2194, Japan; Institute for Advanced Biosciences, Keio University, 403-1 Nipponkoku, Daihouji, Tsuruoka, Yamagata 997-0017, Japan; Humanome Lab, Inc., 2-4-10, Tsukiji, Chuo-ku, Tokyo 104-0045, Japan; Department of Evolutionary Biology and Environmental Studies, University of Zurich, Winterthurerstrasse 190, 8057 Zurich, Switzerland; Kihara Institute for Biological Research, Yokohama City University, 641-12 Maioka, Totsuka-ward, Yokohama, Kanagawa 244-0813, Japan

## Abstract

Although allopolyploid species are common among natural and crop species, it is not easy to distinguish duplicated genes, known as homeologs, during their genomic analysis. Yet, cost-efficient RNA sequencing (RNA-seq) is to be developed for large-scale transcriptomic studies such as time-series analysis and genome-wide association studies in allopolyploids. In this study, we employed a 3′ RNA-seq utilizing 3′ untranslated regions (UTRs) containing frequent mutations among homeologous genes, compared to coding sequence. Among the 3′ RNA-seq protocols, we examined a low-cost method Lasy-Seq using an allohexaploid bread wheat, *Triticum aestivum*. HISAT2 showed the best performance for 3′ RNA-seq with the least mapping errors and quick computational time. The number of detected homeologs was further improved by extending 1 kb of the 3′ UTR annotation. Differentially expressed genes in response to mild cold treatment detected by the 3′ RNA-seq were verified with high-coverage conventional RNA-seq, although the latter detected more differentially expressed genes. Finally, downsampling showed that even a 2 million sequencing depth can still detect more than half of expressed homeologs identifiable by the conventional 32 million reads. These data demonstrate that this low-cost 3′ RNA-seq facilitates large-scale transcriptomic studies of allohexaploid wheat and indicate the potential application to other allopolyploid species.

## INTRODUCTION

RNA sequencing (RNA-seq) is commonly used to study gene expression (1). High-throughput and cost-efficient methods of transcriptomic analysis facilitate large-scale gene expression studies, including time-series analysis and gene-wide association studies, to address diverse biological questions (2–8). For example, Nagano *et al.* (5) and Kashima *et al.* (6) analyzed 2-year time-series transcriptome data using RNA-seq and environmental data in natural fields, which upscaled previous studies using real-time polymerase chain reaction or microarray analysis (9–11), demonstrating plants’ adaptation to seasonal environments. Furthermore, RNA-seq data of a large number of genotypes have been used to detect selection on gene expression. Kremling *et al.* (3) analyzed hundreds of maize RNA-seq samples and showed that the rare alleles were associated with the dysregulation of gene expression. In addition, the pattern of natural selection was reported in rice gene expression using RNA-seq (8). Further large-scale studies are expected to elucidate the interplay between gene functions and adaptations, leading to an improved understanding of the natural species and genetic improvement in the crops.

Cost-efficient methods are critical to facilitate such large-scale RNA-seq experiments. The cost of RNA-seq can be reduced depending on the objective of the experiment, for example, by reducing the sequence coverage for a time-series study, as high coverage is not crucial (5). RNA-seq library synthesis methods based on the 3′-end mRNA sequencing (called 3′ RNA-seq) are innovative methods for cost-efficient sequencing. A typical mRNA consists of a 5′ untranslated region (UTR), coding sequences (CDS), 3′ UTR and a poly(A) tail. 3′ RNA-seq reads encompass the poly(A) tail and the 3′ UTR sequence as well as the CDS if its 3′ UTR is short. Lasy-Seq (12) and BRB-seq (13) are among the current common 3′ RNA-seq methods, with even newer methods in development (14). For Lasy-Seq, the cost per sample, including library preparation and sequencing, can be an order of magnitude lower than that of conventional sequencing methods, when the library is multiplexed at an appropriate scale (12). Such low-cost sequencing protocols facilitate the analysis of not only high-frequency or long-term time-series data, but also species with large and complex genomes.

Polyploid species are widespread in animals, fungi and plants. Approximately one-third of the plant species are estimated to be relatively recent polyploids (15), and crop species tend to be among these (16). Most polyploid species are considered allopolyploid species, derived from the hybridization of different species and genome duplications. Gene copies duplicated by allopolyploidization are called homeologs. Despite the prevalence and importance of polyploid species, genomic studies on polyploids are much less advanced compared to diploid species. The genome size and gene number of allotetraploid and allohexaploid species are double and triple of their diploid parent, respectively, thus increasing the cost of sequencing. Furthermore, due to the high sequence similarity between CDS of homeologs, it is a challenge to analyze homeologous sequences separately in genome assembly and gene expression analysis.

Recent advances in sequencing and bioinformatics technology enabled high-quality genome assemblies of allopolyploid species, most notably of the allohexaploid bread wheat, *Triticum aestivum* (2*n* = 6*x* = 42; AABBDD), with a genome size of ∼16 Gb (17–19). Furthermore, RNA-seq workflows, termed subgenome classification methods, facilitated the use of high-quality genome assemblies of allopolyploid species for high-quality transcriptome analysis (20–23). Moreover, benchmark studies have shown that, when RNA-seq software developed for diploid species, such as STAR (24) or Kallisto, are applied to conventional sequence data of allopolyploid species, the mapping error rate may be as high as >10% depending on species, read length, etc. (21,22). In contrast, subgenome classification methods, including HomeoRoq (25), PolyCat (26) and EAGLE-RC (21), improve the mapping accuracy of allopolyploid species data by comparing the mapping quality on each subgenome to judge its origin.

Recent RNA-seq studies of allopolyploids, including *Arabidopsis*, *Cardamine* and wheat relatives, have highlighted the importance of differential *cis*-regulation of homeologs for environmental responses (23). In model allopolyploid species, *Arabidopsis kamchatica* and *Cardamine flexuosa*, a small percentage of homeologous pairs had altered expression ratio in response to cold, zinc, dry or submergence stress, while the vast majority of pairs maintained the ratio (25,27–29). The *cis*-regulatory differences were inherited from the parental species and can contribute to a generalist niche or environmental robustness by combining parental adaptive traits (23). Bread wheat, *T. aestivum* (2*n* = 6*x* = 42; AABBDD), was formed by allopolyploidization among three species, namely *Triticum urartu* (2*n* = 2*x* = 14; AA), an unknown species related to *Aegilops speltoides* (2*n* = 2*x* = 14; SS) and *Aegilops tauschii* (2*n* = 2*x* = 14; DD) (17–19). The majority of triad homeologs showed balanced expression; however, expression asymmetries are common along wheat chromosomes (30). The time-series transcriptome data were analyzed using the triploid, *Cardamine insueta*, a classic example of contemporary speciation during the past 150 years (31). Although only nine time-series samples were analyzed, the results suggested that *cis*-regulation of key stem cell genes, including *SHOOTMERISTEMLESS*, was inherited from the diploid parental species, *Cardamine rivularis*, and conferred clonal propagation, which is a typical characteristic of polyploid species (31).

The aforementioned studies revealed the difference between the transcriptomic patterns of diploid and polyploid species and premised the importance of large-scale or long-term time-series expression studies of allopolyploid species. To realize such studies, it would be necessary to employ high-throughput and cost-efficient methods that can cope with highly similar homeologous sequences.

Genetic variations, such as single-nucleotide polymorphisms (SNPs) and insertions and deletions (indels), are higher in 3′ UTR than in the CDS of homeologous sequences (32–35) because stronger negative selection of CDS reduces its polymorphisms. Thus, owing to high sequence divergence in the 3′ UTR, the ratio of unique mapping in allopolyploid species is expected to be high, even without employing subgenome classification methods. In this study, we tested the homeolog expression analysis by using Lasy-Seq to exploit the high genetic variations in the 3′ UTR. Using the allohexaploid species, *T. aestivum*, we first examined the accuracy of subgenome mapping by using the RNA-seq data of Tetra Chinese Spring (TCS) as a ground truth. In the TCS, the subgenome D was removed from an experimental hexaploid cultivar, Chinese Spring (CS), by repeated backcrossing (36); thus, the AB subgenomes of TCS must be very close to the AB subgenomes of CS. Therefore, the mapping rate of RNA-seq data of TCS on the subgenome D can be used as a proxy of mapping error rate (21,22). Thereafter, we improved mapping rates by modifying the incomplete gene annotations of the reference genome. This method was then applied to detect differentially expressed genes (DEGs) in *T. aestivum* after a mild cold treatment. Lastly, we examined the effect of reducing sequence reads on the detection of homeolog expression patterns. Collectively, the results suggest that low-coverage 3′ RNA-seq can be useful for large-scale transcriptomic studies of allohexaploid species wheat.

## MATERIALS AND METHODS

### Reference sequence and annotation of wheat

Reference sequences of allohexaploid wheat, CS, and its annotations were downloaded from the International Wheat Genome Sequencing Consortium (IWGSC) website (17). Reference sequence v1.0 and annotation v1.1 were used in this study. Reference sequence v2.1 was not used because (i) the update in v2.1 was considered relatively minor and (ii) the 3′ UTR annotation of both the versions is incomplete. Furthermore, (iii) gene ontology (GO) is available on annotation v1.0, and it can be easily converted to v1.1 by simply replacing the version number in the gene ID. For instance, in v1.0 annotation, TraesCS7A01G115400 would be equivalent to TraesCS7A02G115400 in v1.1. However, we extended the gene annotation in the original IWGSC annotation version 1.1. The annotation of each gene was uniformly extended by 0.5, 1.0, 1.5, 2.0, 2.5, 3.0, 3.5 and 4.0 kb. If the extended region overlapped with the downstream gene on the same strand, the extension was stopped before the next gene. Both IWGSC and extended IWGSC annotations were used in this study. In addition, the reference sequence of chloroplast DNA with the accession number NC_002762.1 was downloaded from GenBank to check the mapping coverage of chloroplast DNA.

### Identification of homeologous regions and homeolog triads

The homeolog triad list was prepared following a previous study (21). We extracted the transcript sequences of high-confidence genes from A, B and D subgenomes, based on the IWGSC CS reference sequence v1.0 and annotation v1.1. Homeolog pairs in the AB, AD and BD subgenomes were defined as pairs that were reciprocal best hits by mapping transcripts from one subgenome to the other two using LAST v950 (37). Homeolog triads were defined as homeologs of AB subgenomes that also shared the same hit for D in their AD and BD pairs. Since we focused on the mapping to each subgenome, we did not include genomic sequences with unknown chromosomal location (i.e. the gene accession number starting with ChrUn).

### Plant materials and RNA-seq library preparation

We grew three individuals of CS for RNA-seq. All CS plants for RNA sampling were grown in a plant growth chamber KCLP-1000II-CT (Nippon Medical & Chemical Instruments Co., Ltd, Osaka, Japan) for 5 weeks at 22°C, and the fourth leaves were sampled. Afterward, the CS plants were kept in a walk-in growth chamber in Kihara Institute of Biological Research (Kanagawa, Japan) for 1 week at 7°C followed by sampling of the fifth leaves thereafter considered as cold-treated samples. Both the growth chambers were set at 14/10 h light/dark cycle using time-controlled white fluorescent light. The tip of the fully expanded youngest leaf of each individual was sampled and immediately soaked into RNA stabilization solution, RNA*later* (Thermo Fisher Scientific Inc., Waltham, MA), and the samples were stored in a biomedical freezer. From these samples, the total RNA was extracted using the RNeasy Plant Mini Kit (QIAGEN, Venlo, The Netherlands) according to the manufacturer’s instructions.

The TCS plants were grown in the experimental field of Kihara Institute of Biological Research. The seedlings were planted there on 9 December 2018. The leaves were sampled on 30 December 2018 and 26 April 2019 for three samples each. The field climate record showed that the daily lowest, mean and highest air temperatures in the last week were 6.8, 11.5 and 17.2°C for the first sampling, and 14.4, 18.2 and 23.5°C for the second sampling, respectively. TCS leaves were sampled and stored in a similar manner to the above CS leaf samples. The RNA stabilization solution was homemade nucleic acid preservation medium (0.014 M ethylenediaminetetraacetic acid disodium, 0.018 M trisodium citrate, 3.8 M ammonium sulfate and H_2_SO_4_ to adjust pH 5.2). The total RNA was extracted using the Maxwell^®^ RSC Plant RNA Kit with Maxwell^®^ RSC Instrument (Promega Corp., Madison, WI) according to the manufacturer’s instructions.

For each of the six CS RNA samples, a portion was used to prepare 3′ RNA-seq library with the Lasy-Seq v1.0 protocol that gives strand-specific data (25), and another portion was used to prepare conventional paired-end RNA-seq library with the TruSeq Stranded mRNA Library Prep Kit (Illumina, San Diego, CA). All the CS libraries were sequenced using Illumina HiSeq 4000. RNA libraries for the six TCS RNA samples were prepared with the Lasy-Seq v1.0 protocol. All the TCS libraries were sequenced using Illumina HiSeq X.

The 3′ RNA-seq libraries were sequenced as paired-end reads (12). Read 1 of the paired-end reads contains mRNA sequence and poly(A), while read 2 contains unique molecular identifiers, sample index and poly(T) derived from the primer and template RNA. Thus, only read 1 was used for the downstream analysis.

### Preprocessing of RNA-seq reads

The 3′ RNA-seq reads of the CS and TCS samples were preprocessed using Trimmomatic v0.36 (38) to (i) remove the first 10 bases from the 5′ end of the reads, as the base appearance frequency was not random, and (ii) trim low-quality bases over the whole reads. Thereafter, poly(A) and poly(T) were trimmed from the 3′ end using a custom script, since Lasy-Seq results in a large proportion of poly(A) on the 3′ end of the reads. Finally, reads >40 bases were passed for downstream analyses.

The conventional RNA-seq reads of the CS samples were preprocessed using Trimmomatic v0.36 to trim low-quality bases over the whole reads. Only reads >40 bases were passed for downstream analysis.

### Homeolog expression quantification for 3′ RNA-seq samples

Homeolog expression of 3′ RNA-seq samples (i.e. the six CS and TCS samples) was quantified using HISAT2 (39), STAR (24) and EAGLE-RC (21). To quantify homeolog expression using HISAT2 and STAR, we mapped the preprocessed reads of 3′ RNA-seq onto the IWGSC CS reference sequence v1.0 using HISAT2 v2.2.1 with ‘--no-spliced-alignment’ option and STAR v2.5.3a with ‘--alignIntronMax 1 --outMultimapperOrder Random’, respectively. We then counted the reads that were mapped on gene regions using the IWGSC and extended IWGSC annotations with featureCounts v1.6.0 (40). We also counted the reads that were uniquely mapped on each subgenome with SAMtools (41). To quantify homeolog expression using EAGLE-RC, we followed the standard protocol in which (21) we (i) separately mapped reads onto A, B and D subgenomes of the IWGSC CS reference sequence v1.0 with STAR v2.5.3a, (ii) identified SNPs with EAGLE (42) by considering homeologous regions, (iii) classified homeolog read origins with EAGLE-RC and (iv) counted the reads that were mapped on the homeologous region using featureCounts v1.6.0 with the extended IWGSC+1k annotation.

### Homeolog expression quantification for conventional RNA-seq

The preprocessed reads of conventional RNA-seq samples were processed using EAGLE-RC following the standard procedure (21), similar to that employed for 3′ RNA-seq samples. The number of reads mapped to the gene region was counted using featureCounts with IWGSC+1k annotation.

### Differential expression analysis

The six CS samples consisted of three samples that were subjected to 7°C for 7 days and three control samples harvested before the treatment from the same individuals. For both 3′ RNA-seq and conventional RNA-seq, gene expression was quantified by HISAT2. Then, we detected genes differentially expressed between the cold and control treatments using likelihood ratio test packaged in edgeR v3.39.6 (43). As the control and cold-treated tissues were sampled from the same individuals, the design matrix for the likelihood ratio test was created by considering pairwise comparison. Genes were identified as DEGs at false discovery rate (FDR) <0.05 and the absolute value of log_2_ fold change (|log_2_FC|) >1. GO enrichment analysis was performed against the DEGs using topGO v2.49.0 (44).

### Downsampling analysis

We downsampled the reads of the six 3′ RNA-seq samples in the CS dataset with downsampling rates of 0.8, 0.6, 0.4 and 0.2. A total of 6 × 4 = 24 downsampled samples were processed, and the homeolog expression was quantified using HISAT2 following the same procedures used for processing the original dataset. Genes were defined as ‘detected’ if one or more reads mapped onto the gene region. Similarly, a homeolog triad was defined as ‘detected’ if one or more reads mapped onto the homeologous regions on at least one of the A, B and D subgenomes. Note that a homeolog triad was counted as three genes only if all the three homeologs were expressed; however, if only one or two homeologs were expressed, it was counted as one or two genes. In addition, if it is a subgenome-specific gene, it was counted as one gene but not as a homeolog triad. Thus, the number of genes is about three times the number of homeologous triads, but not exactly three times.

### Computational resources

Read preprocessing and read classification were performed on a supercomputer with Intel^®^ Xeon^®^ CPU E5-2630 v4 @ 2.20 GHz and 125 GB memory. The execution time was measured using these computational resources.

## RESULTS AND DISCUSSION

### Homeolog triads

Based on a technique used in a previous study (21), we identified 19 856 homeolog triads (Supplementary Data 1) by using a LAST-based homology search. This criterion identified more triads than the IWGSC’s triads (18 407 triads) (30). Among them, we found there are 16 606 triads shared by both triad lists. Additionally, when we focused on the triads with >0 read counts in at least one of the six CS samples sequenced by the conventional RNA-seq, there were 14 226 and 12 617 triads identified in this study and the IWGSC’s triad lists, respectively, of which 12 508 triads were shared by both lists. Considering the high proportion of homeolog triads common to both lists, and aiming for a comparable analysis (21), we used our list that includes more triads in this study.

### Performance evaluation of read assignment

The original report using Lasy-Seq by Nagano *et al.* (5) analyzed relatively low read numbers for cost efficiency (∼4.2 million reads). To evaluate the applicability of these methods for polyploid species, six CS samples were sequenced using 3′ RNA-seq, and on an average, 3.0 million single-end reads with 126 bases per library were sequenced. These sequence reads were then preprocessed by removing the adapter sequences, low-quality bases and poly(A) tails. As a result, on an average, 77.4 ± 0.8% reads remained after preprocessing (Supplementary Data 2, Sheet 1).

We mapped the preprocessed reads onto the IWGSC CS reference sequence v1.0 using both the HISAT2 and STAR. In RNA-seq analysis of allopolyploid species, reads that are uniquely assigned are informative in quantifying the expression ratio of homeologs, while reads that are mapped to sequences identical between homeologs can be proportionally added to infer absolute expression level (27). Consistent with high divergence of 3′ UTR, we found that 59.0 ± 1.7% and 61.5 ± 1.5% of the preprocessed reads were uniquely mapped on one of the A, B and D subgenomes using HISAT2 and STAR, respectively. Among them, we found that the proportions of such reads mapped to each of the three subgenomes were nearly identical—close to one-third (Table 1). Besides nuclear genome, we found that 12.4% of the preprocessed reads were uniquely mapped onto the chloroplast DNA with HISAT2 (Supplementary Data 2, Sheet 2). For EAGLE-RC, in contrast to alignment-based methods, only 14.2 ± 0.8% of the preprocessed reads were usable for read assignment. Since EAGLE-RC only considers reads mappable on all the subgenomes, the high portion of unique mapping made EAGLE-RC too conservative to analyze the 3′ RNA-seq data that encompass a high rate of polymorphisms.

**Table 1.** The proportion of preprocessed reads and reads that were further classified to have A, B and D subgenome origins against CS samples sequenced by 3′ RNA-seq

Classification tool	% Reads attributable to each subgenome	% Reads classified as A	% Reads classified as B	% Reads classified as D	Time (min/1 million reads)
HISAT2	59.0 ± 1.7	33.2 ± 0.3	33.5 ± 0.3	33.3 ± 0.2	0.3 ± 0.1
STAR	61.5 ± 1.5	33.2 ± 0.3	33.5 ± 0.2	33.3 ± 0.2	0.8 ± 0.4
EAGLE-RC	14.2 ± 0.8	32.4 ± 0.7	35.7 ± 0.9	31.9 ± 0.4	35.4 ± 13.2

Average ± standard deviation values were calculated from six samples. The second column (i.e. % reads attributable to each subgenome) indicates the proportion of the preprocessed reads that were successfully assigned to one of the three subgenomes. Among them, reads classified to subgenomes A, B and D are shown in the second, third and fourth columns, respectively (summed to 100%). The last column shows the calculation time.

The computation times for read classification were considerably different between the alignment-based methods (HISAT2 and STAR) and classification-based method (EAGLE-RC). HISAT2 and STAR took ∼0.3 and ∼0.8 min to process a million reads with 16 computational threads, respectively. In contrast, EAGLE-RC took ∼35.4 min to process a million reads with 16 threads because it involves multiple processes, including subgenome-independent mapping, SNP calling, homeolog likelihood calculation and read classification.

To evaluate the classification error rates of 3′ RNA-seq, we also sequenced six TCS samples using 3′ RNA-seq: ∼2.6 million single-end reads with 151 bases per library were sequenced. Thereafter, the reads were preprocessed and mapped using the same procedures used for analyzing CS 3′ RNA-seq samples. There were 81.8 ± 2.1% reads left after the preprocessing (Supplementary Data 3, Sheet 1). We then mapped and classified homeolog reads using the HISAT2, STAR and EAGLE-RC protocols, and 60.5 ± 1.9%, 64.4 ± 1.9% and 18.0 ± 1.4% of the preprocessed reads were assigned as those that mapped on one of the subgenomes by HISAT2, STAR and EAGLE-RC, respectively; among them, most reads were assigned to A or B subgenome and only 3.2 ± 0.3%, 3.4 ± 0.4% and 2.2 ± 0.3% were assigned to D subgenome by HISAT2, STAR and EAGLE-RC, respectively (Table 2). Since TCS was generated by removing the D subgenome from the CS, mapping rate on D subgenome can be used as a proxy of mapping error rate (21). All three methods had a relatively low misclassification rate. Among the alignment-based methods, HISAT2 showed a slightly lower error rate than STAR.

**Table 2.** The proportion of preprocessed reads and reads that were further classified to have A, B and D subgenome origins against TCS samples sequenced by 3′ RNA-seq

Classification tool	% Reads attributable to each subgenome	% Reads classified as A	% Reads classified as B	% Reads classified as D (error rate)	Time (min/1 million reads)
HISAT2	60.5 ± 1.9	49.9 ± 0.1	47.0 ± 0.3	3.2 ± 0.3	2.4 ± 0.3
STAR	64.4 ± 1.9	49.8 ± 0.1	46.9 ± 0.3	3.4 ± 0.4	1.8 ± 0.5
EAGLE-RC	18.0 ± 1.4	48.4 ± 0.8	49.4 ± 0.6	2.2 ± 0.3	39.5 ± 8.2

Average ± standard deviation values were calculated from six samples. The second column (i.e. % reads attributable to each subgenome) indicates the proportion of the preprocessed reads that were successfully assigned to one of the three subgenomes. Among them, reads classified to subgenomes A, B and D are shown in the second, third and fourth columns, respectively (summed to 100%). The last column shows the calculation time. Because TCS lacks D subgenome, reads mapped on D subgenome are recognized as classification error.

In summary, among these analyses, HISAT2 and STAR produced similar proportions of reads attributable to each subgenome, whereas EAGLE-RC was not suitable for 3′ RNA-seq. In addition, both HISAT2 and STAR produced low proportions of misclassified reads; however, HISAT2 had a lower rate. Moreover, HISAT2 is ∼2.7 times faster than STAR in computing the hexaploid data and 118 times faster than EAGLE-RC. Thus, we concluded that HISAT2 is most suitable for the 3′ RNA-seq data and used it for subsequent analysis.

### Effect of the annotation on the quantification of homeolog expression

Subsequently, we examined the effect of annotation extension in the 3′ regions, since many genes (homeologs) only had annotations of the coding region in the IWGSC annotation. This is presumably because homology to known genes is informative to predict coding sequences but not effective in noncoding regions with higher divergence. Although full-length complementary DNA data can provide strong evidence to annotate 3′ UTRs, the quality of such data may be limited in allopolyploid species.

In RNA-seq, relative expression levels can be considered as the number of reads mapped on the gene region. However, due to the insufficiency of the 3′ UTR annotations in the IWGSC annotation, counting the mapped reads in the 3′ RNA-seq samples based on this annotation may result in underestimation of the expression levels. It was reported that the extension of the 3′-end annotation of each gene model can improve RNA-seq analysis in barley, as many predicted gene models lacked 3′ UTR regions in this species (45). To address this issue, we extended the gene region by 0.5–4.0 kb longer than the IWGSC annotation and counted the reads assigned to both the original and extended gene regions.

**Figure 1.**
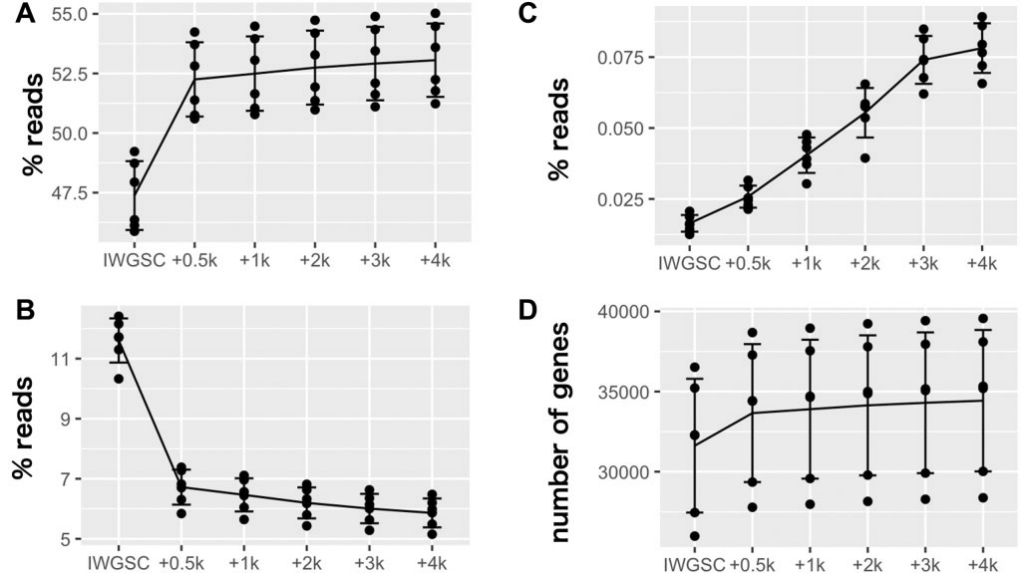
Effects of extending the 3′-end annotation of IWGSC. (**A**) The proportion of uniquely mapped reads that were assigned to the annotated gene regions. (**B**) The proportion of reads that were uniquely mapped on the reference sequence without annotation. (**C**) The proportion of reads that were mapped on multiple annotated gene regions. (**D**) The number of genes detected with each annotation.

To investigate the effect of annotation extension, we compared the read counts assigned to the original gene regions and the extended gene regions by HISAT2 using CS 3′ RNA-seq samples. We found that the annotation extension of the gene regions increased the number of reads assigned to that region (Figure 1 and Supplementary Data 4). With an extension of 0.5 kb, the increase of the number of assigned reads was 4.9%; however, this declined for extensions longer than 0.5 kb. For example, the number of assigned reads increased only 0.2% by an increase of extension from 0.5 to 1.0 kb.

For further analysis, we used the IWGSC annotation with 1.0 kb extension (IWGSC+1k). By using this annotation, 53.2% reads were mapped on the annotated gene region, which is 5.2% more than the original IWGSC annotation (48.0%) (Figure 1A); in contrast, 6.6% reads were mapped on the unannotated gene region, which is 5.2% less than the original IWGSC annotation (11.8%) (Figure 1B). In addition, extending the annotation may result in overlapping between two adjacent genes. We found that 0.04% of the reads mapped on the overlapped regions with the IWGSC+1k annotation, which was only 0.02% higher than the original IWGSC annotation (0.02%) (Figure 1C). Those reads mapped on the overlapped regions were excluded from the downstream analysis due to the difficulty in exact assignment of the origin. Furthermore, 33 899 genes were detected with the IWGSC+1k annotation, which was 2276 more than the number of genes detected with the original IWGSC annotation (31 623) (Figure 1D).

**Figure 2.**
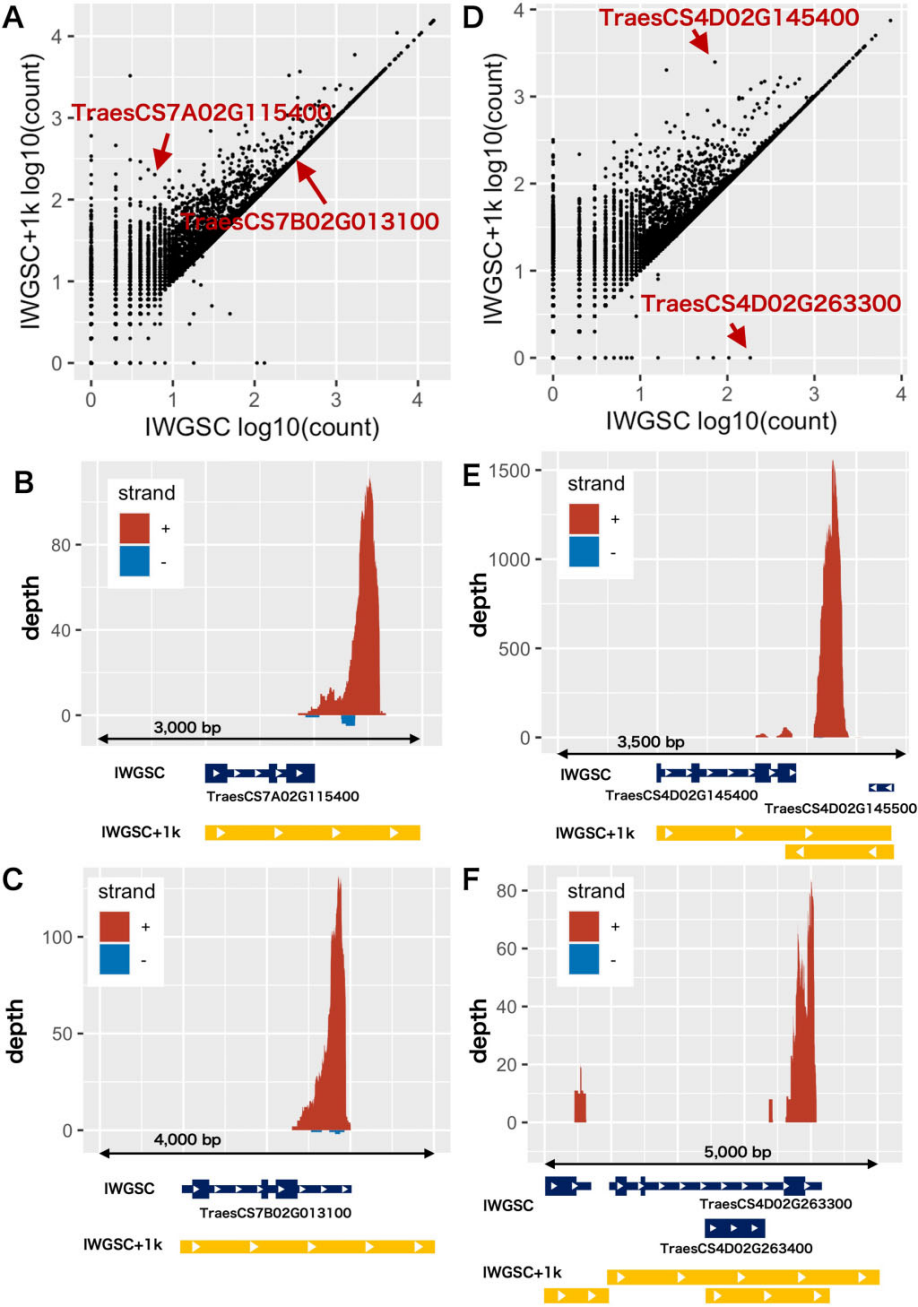
The number of read counts assigned to gene regions in TCS and CS samples. Scatter plots showing the correlation between the numbers of read counts that were assigned to gene regions annotated with the IWGSC and IWGSC+1k annotations for the TCS sample (**A**) and the CS sample (**D**). The actual mapping coverage (i.e. depth) of the two genes TraesCS7A02G115400 and TraesCS7B02G013100, indicated by the red arrows in panel (A), is shown in panels (**B**) and (**C**), respectively, while mapping coverage of the two genes TraesCS4D02G145400 and TraesCS4D02G263300 indicated in panel (D) is shown in panels (**E**) and (**F**), respectively. For panels (B), (C), (E) and (F), the *y*-axis represents the mapping coverage; the coverage in the forward and reversed strands to the reference sequence is shown in red and blue, respectively; and gene regions annotated with IWGSC and IWGSC+1k are shown in dark blue and orange, respectively.

To investigate the details of read counting with and without annotation extension, we additionally compared the read counts (i.e. gene expression) obtained from the IWGSC and IWGSC+1k annotations for each gene in one of the TCS 3′ RNA-seq samples. We found that 29 282 genes (81.5% of the expressed genes) showed the same expression levels between the two annotations, while 6579 genes (18.3%) had increased read counts and only 44 genes had decreased read counts when using IWGSC+1k annotation (Figure 2A), suggesting the benefit of extension. For further analysis, we focused on two genes with different or same read counts between the IWGSC and IWGSC+1k annotations. We found that by extending the annotations, the expression of some genes relevant to important phenotypes, such as TraesCS7A02G115400, which is known as *FT1-A1*/*VRN-A3* encoding florigen to control heading time, could be better quantified (Figure 2B). Not surprisingly, for genes such as TraesCS7B02G013100, which has good 3′ UTR annotation in IWGSG, extending the annotations did not influence the read counting (Figure 2C), confirming that the IWGSC annotation is adequate for this gene.

In addition, we checked the annotation extending effects on the CS 3′ RNA-seq samples (Figure 2D). The trends for the CS samples were similar to those for the TCS samples. Here, we illustrate two examples in which the extended 3′ UTR of one gene overlapped with the region of an adjacent gene. The first example shows that the extended 3′ end overlaps between two genes with opposite strands (Figure 2E). Since reads sequenced by 3′ RNA-seq are strand-specific, reads mapped on the overlapped regions can be distinguished by the mapping strand. In this case, reads that mapped on forward strand were counted for TraesCS4D02G145400 but not for TraesCS4D02G145500. As a result, the read count of TraesCS4D02G145400 with the IWGSC+1k annotation was more than that with the IWGSC annotation. Another example shows that the extended 3′ end overlaps between two genes with the same strand (Figure 2F). By extension, TraesCS4D02G263300 and TraesCS4D02G263400 partially share the 3′ UTR region. Because the reads mapped on the regions with multiple annotations are not considered due to the uncertainty, the read count of TraesCS4D02G263300 decreased in the IWGSC+1k annotation. On the other hand, the counts of TraesCS4D02G263400 remain zero and are not plotted in the figure.

Here, we have shown that 3′ RNA-seq could be rather largely influenced by the quality of gene annotations, as it focuses on the 3′ end of the mRNA. Most gene annotations prioritize CDS regions, so that the quality of UTR annotations is poor. We also demonstrated that gene detection could be improved by extending the insufficient annotation of the 3′ end, similar to a study using barley (45). The number of detected genes increased by nearly 10% in this approach (Figure 1 and Supplementary Data 4), showing a wider application possibility for the species with incomplete UTR annotation.

### Correlation of homeolog expression levels between 3′ RNA-seq and conventional RNA-seq methods

3′ RNA-seq and conventional RNA-seq methods are based on different experimental procedures of library synthesis and do not necessarily give equivalent expression levels. It is known that Pearson’s correlation coefficient of gene expression levels is relatively high (0.88 between 3′ RNA-seq and conventional RNA-seq) although low-expression genes tend to show low correlation (12,46), implying that the expression changes should be quantified using a dataset generated by a single method.

To examine the correlation in the polyploid species, we sequenced the same CS RNA samples using conventional RNA-seq as another library and sequencing protocol, in addition to the 3′ RNA-seq data described above. Approximately 32.0 million read pairs with 151 × 2 bases per library were obtained by a conventional RNA-seq protocol, and the reads were preprocessed using the same procedures used for analyzing CS 3′ RNA-seq samples. Then, we quantified the expression of CS conventional RNA-seq samples using two methods, HISAT2 and EAGLE-RC (Supplementary Data 5). The former is the same as the methods used for the quantification of the 3′ RNA-seq data, and also confirmed the expectation of high unique mapping ratio to the A, B or D subgenome of the 3′ RNA-seq data (59.0% in contrast to 39.7% of conventional RNA-seq) owing to frequent SNPs and indels in 3′ UTR (Supplementary Data 2 and 5). The latter is known to reduce mismapping of conventional RNA-seq data by excluding unclassifiable reads (21). During the quantification process, we counted the reads in each homeolog region using the IWGSC+1k annotation. For 3′ RNA-seq, we normalized the raw read counts to counts per million reads (CPM) because normalization by gene length is unnecessary with sequences only at the 3′ end of mRNAs. For conventional RNA-seq, the raw read counts were normalized to transcripts per million (TPM) by normalizing homeolog length and total number of reads, considering that longer transcripts result in more reads. We then calculated the Pearson’s correlation coefficients of log_10_-transformed normalized expression of expressed genes (i.e. CPM > 0 for 3′ RNA-seq samples and TPM > 0 for conventional RNA-seq samples) between 3′ RNA-seq (quantified with HISAT2) and conventional RNA-seq (quantified with HISAT2 and EAGLE-RC).

**Figure 3.**
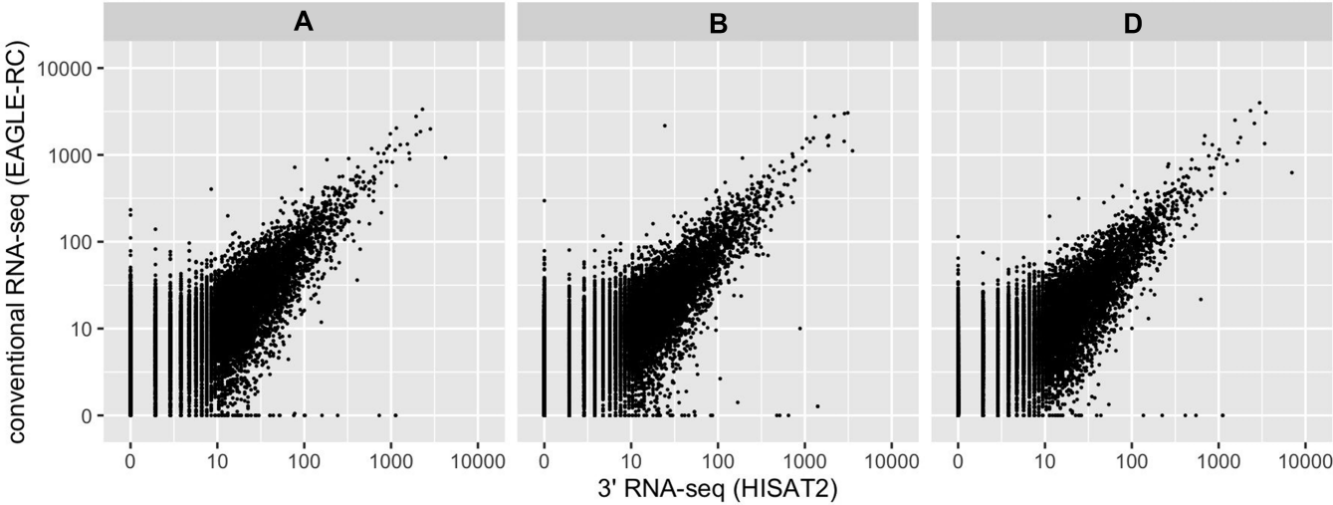
Correlation of expression levels of A, B and D homeologs between the 3′ RNA-seq quantified by HISAT2 and conventional RNA-seq quantified by EAGLE-RC of a CS sample.

We found that the average values of correlation coefficients of six samples for A, B and D homeologs between 3′ RNA-seq quantified with HISAT2 and conventional RNA-seq quantified with EAGLE-RC were 0.73 ± 0.05, 0.74 ± 0.05 and 0.74 ± 0.05, respectively (Figure 3 and Supplementary Data 6). A similar result was obtained between 3′ RNA-seq quantified with HISAT2 and conventional RNA-seq quantified with HISAT2 (0.75 for A, B and D subgenomes), which is consistent with high correlation of the quantification of conventional data using HISAT2 and EAGLE-RC (0.97–0.98) (Supplementary Data 6). This suggests that the choice of HISAT2 and EAGLE-RC contributed little to the level of correlation.

In addition, we made ternary plots to visualize the expression ratio of the A, B and D homeologs (30) using 3′ RNA-seq and conventional RNA-seq. Most genes are located toward the middle of the triangle despite the difference in the number of detected homeologs, suggesting that the balanced expression level of the A, B and D homeologs can be detected by both 3′ RNA-seq and conventional RNA-seq (Supplementary Figure S1).

These analyses suggest that the expression levels of the allohexaploid wheat quantified by 3′ RNA-seq are correlated with the one with conventional RNA-seq although the difference between the methods is more pronounced than in the diploid *A. thaliana* (12). We speculate that the different library synthesis protocols (i.e. 3′ RNA-seq and conventional RNA-seq) contributed to the difference in quantification results because the choice of HISAT2 (the same as 3′ RNA-seq data) and EAGLE-RC in conventional RNA-seq had little effect on correlation. A standard conventional RNA-seq library synthesis protocol generates short fragments from the whole mRNA. In contrast, a 3′ RNA-seq library synthesis protocol generates one fragment from one mRNA, targeting the 3′ end. Further studies would be necessary to reveal relative contribution of different factors for the differences.

### DEGs analyzed by 3′ RNA-seq

We next compared DEGs detectable by the low-coverage 3′ RNA-seq with those by the conventional RNA-seq data. There were 218 and 4806 genes differentially expressed between mild cold treatment and control CS samples detected from the low-coverage 3′ RNA-seq and conventional RNA-seq data quantified using HISAT2 with the criteria of FDR < 0.05 and |log_2_FC| > 1, respectively (Supplementary Data 7). We found that a vast majority of DEGs of 3′ RNA-seq data (92.7%) were confirmed by the conventional RNA-seq data (32.0 million reads) (Supplementary Data 7, Sheet 5). This corresponds to 133 triads showing at least one of the three homeologs was detected as DEG by 3′ RNA-seq, and 128 (96.2%) of them were detected by the conventional RNA-seq. Genes associated with cold stress, such as those encoding late embryogenesis abundant protein (*LEA*), dehydrin, ethylene-responsive transcription factors and aquaporin, were identified as DEGs from both 3′ RNA-seq and conventional RNA-seq data. In addition, flowering-promoting factor-1 like protein 1 (*FPFL1*), which involved vegetative–reproductive transition, was also upregulated by cold treatment. These results suggest that although many more significant DEGs can be identified by deeper coverage of conventional RNA-seq, DEGs are also identifiable by low-coverage 3′ RNA-seq methods.

We furthermore conducted GO enrichment analysis, although the wheat GO annotation is unfortunately limited and the GO category ‘response to cold’ is not annotated in IWGSC reference annotation. Yet, we found 13 enriched GOs (*P* < 0.05) (Supplementary Data 7, Sheet 2). Additionally, among the 13 significantly enriched GOs, there were 6 GOs also enriched in DEGs of the conventional RNA-seq data (Supplementary Data 7, Sheet 4) and the other 7 GOs only detected from DEGs of 3′ RNA-seq. Although these seven GOs were not enriched in DEGs of the conventional RNA-seq data, we found that many other similar GOs were enriched (Supplementary Data 7, Sheet 6). Many GO categories related to amino acid metabolism were enriched in both datasets. Amino acids are known as compatible solutes, which are small organic molecules acting as osmoprotectants against cold or other stresses (47). Although little is known about the function of those metabolites in wheat cold response yet, our result implies their possible importance in cold response.

Taken together, 3′ RNA-seq provides a balanced opportunity to obtain biologically significant implications with low cost, although a higher coverage of 3′ RNA-seq might be more powerful in differential analysis of gene expression.

### Sequencing depth and the number of detected genes and homeologs

**Figure 4.**
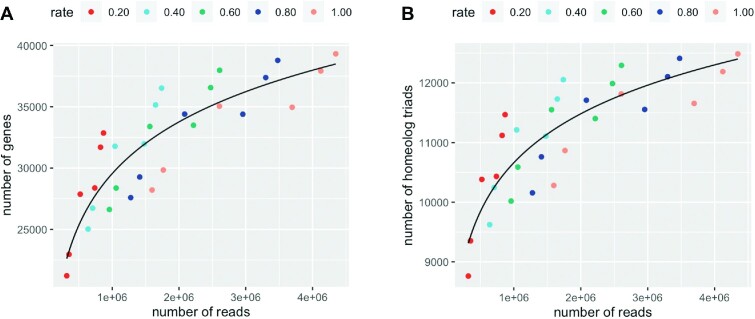
Scatter plot representing the relationship between the sequencing depth in 3′ RNA-seq and the number of detected genes (**A**) and homeolog triads (**B**) for CS samples. The colors indicate the downsampling rates.

To systematically address the effects of sequencing depth on the detection of expressing genes, we performed a downsampling experiment. We created datasets by downsampling 20%, 40%, 60% and 80% of the reads from each original CS 3′ RNA-seq sample and performed gene expression quantification using the same protocol that was applied to the original dataset. The result indicated that the number of additionally detected genes decreased logarithmically with respect to sequencing depth (Figure 4). The number of detected genes was estimated to be 29 282, 33 454, 35 894 and 37 626 for 1.0, 2.0, 3.0 and 4.0 million reads, respectively. At 2.0 million sequencing depth, 33 454 genes and ∼11 482 homeolog triads were expected to be detected. Considering that 61 340 genes (15 726 homeolog triads) were detected in conventional RNA-seq samples by 32.0 million sequencing reads, the detection power of 3′ RNA-seq is quite high as it detected 54.5% of the genes as well as 73.0% homeolog triads with only 2.0 million reads.

## CONCLUDING REMARKS

3′ RNA-seq was developed as a low-cost RNA-seq technology (12). We applied this method to analyze homeolog expression in bread wheat, *T. aestivum*, an allohexapolyploid with relatively large subgenomes. In addition to the usage of cost-efficient Lasy-Seq method, our downsampling results suggested that the coverage of 2 million reads per sample allows >100 multiplexing per lane for many existing sequencing systems. HISAT2 showed rapid computation with relatively low error in mapping. Compared with genes or homeolog triads that were detected with 32.0 million reads by using the conventional RNA-seq data, approximately half of them were detected with only 2 million reads by using 3′ RNA-seq methods. Considering both cost-effectiveness and quality, 3′ RNA-seq should be considered for large-scale transcriptomic, time-series or condition transition analyses of allohexaploid wheat, providing useful insights into breeding as well as environmental adaptation. Even if technical adjustments may be required, 3′ RNA-seq is also expected to contribute to the research of other agriculturally or ecologically important allopolyploid species.

## DATA AVAILABILITY

The RNA-seq data are deposited on DDBJ DRA with the accession number DRA015200. The scripts used in this study are deposited on Zenodo with doi:10.5281/zenodo.7402763.

## Supplementary Material

lqad067_Supplemental_Files
