# Supplementary material for: A low-coverage 3′ RNA-seq to detect homeolog expression in polyploid wheat
Source: NAR Genom Bioinform. 2023 Jul 12;5(3):lqad067. doi: 10.1093/nargab/lqad067 (PMC10336777; doi:10.1093/nargab/lqad067)

Supplementary Information for “A low-coverage 3′ RNA-seq to detect homeolog expression in polyploid wheat”

Jianqiang Sun^1^, Moeko Okada^2,3^, Toshiaki Tameshige^3,4^, Rie Shimizu-Inatsugi^2^, Reiko Akiyama^2^, Atsushi J. Nagano^5,6^, Jun Sese^7^, Kentaro K. Shimizu^2,3,*^

­­

^1^ Research Center for Agricultural Information Technology, National Agriculture and Food Research Organization, 3-1-1 Kannondai, Tsukuba, Ibaraki 305-8517, Japan

^2^ Department of Evolutionary Biology and Environmental Studies, University of Zurich, 8057 Zurich, Switzerland

^3^ Kihara Institute for Biological Research, Yokohama City University, Maioka, Totsuka-ward, Yokohama, Kanagawa 244-0813, Japan

^4^ Division of Biological Sciences, Graduate School of Science and Technology, Nara Institute of Science and Technology, 8916-5, Takayama-cho, Ikoma, Nara, 630-0192, Japan

^5^ Faculty of Agriculture, Ryukoku University, Yokotani 1-5, Seta Ohe-cho, Otsu, Shiga 520-2194, Japan

^6^ Institute for Advanced Biosciences, Keio University, 403-1 Nipponkoku, Daihouji, Tsuruoka, Yamagata, 997-0017, Japan

^7^ Humanome Lab., Inc. 2-4-10, Tsukiji, Chuo-ku, Tokyo, 104-0045, Japan

# Supplementary data

**SI Data 1** (SI_Data_1.xlsx): A list of homeolog triads defined by this study and those of the IWGSC.

**SI Data 2** (SI_Data_2.xlsx): Mapping and quantification statistics of 3′ RNA-seq data of CS samples.

**SI Data 3** (SI_Data_3.xlsx): Mapping and quantification statistics of 3′ RNA-seq data of TCS samples.

**SI Data 4** (SI_Data_4.xlsx): Quantification statistics of 3′ RNA-seq data of CS samples with the extended IWGSC annotations.

**SI Data 5** (SI_Data_5.xlsx): Mapping and quantification statistics of conventional RNA-seq data of CS samples.

**SI Data 6** (SI_Data_6.xlsx): Expression correlations between 3′ RNA-seq and conventional RNA-seq data of CS samples.

**SI Data 7** (SI_Data_7.xlsx): DEGs of 3′ RNA-seq and conventional RNA-seq data of CS samples.

**Figure S1:** Ternary plots show the distribution of expression ratio of homeolog triads quantified with 3′ RNA-seq (HISAT2) and conventional RNA-seq (HISAT2 and EAGLE-RC) under control and mild cold conditions.


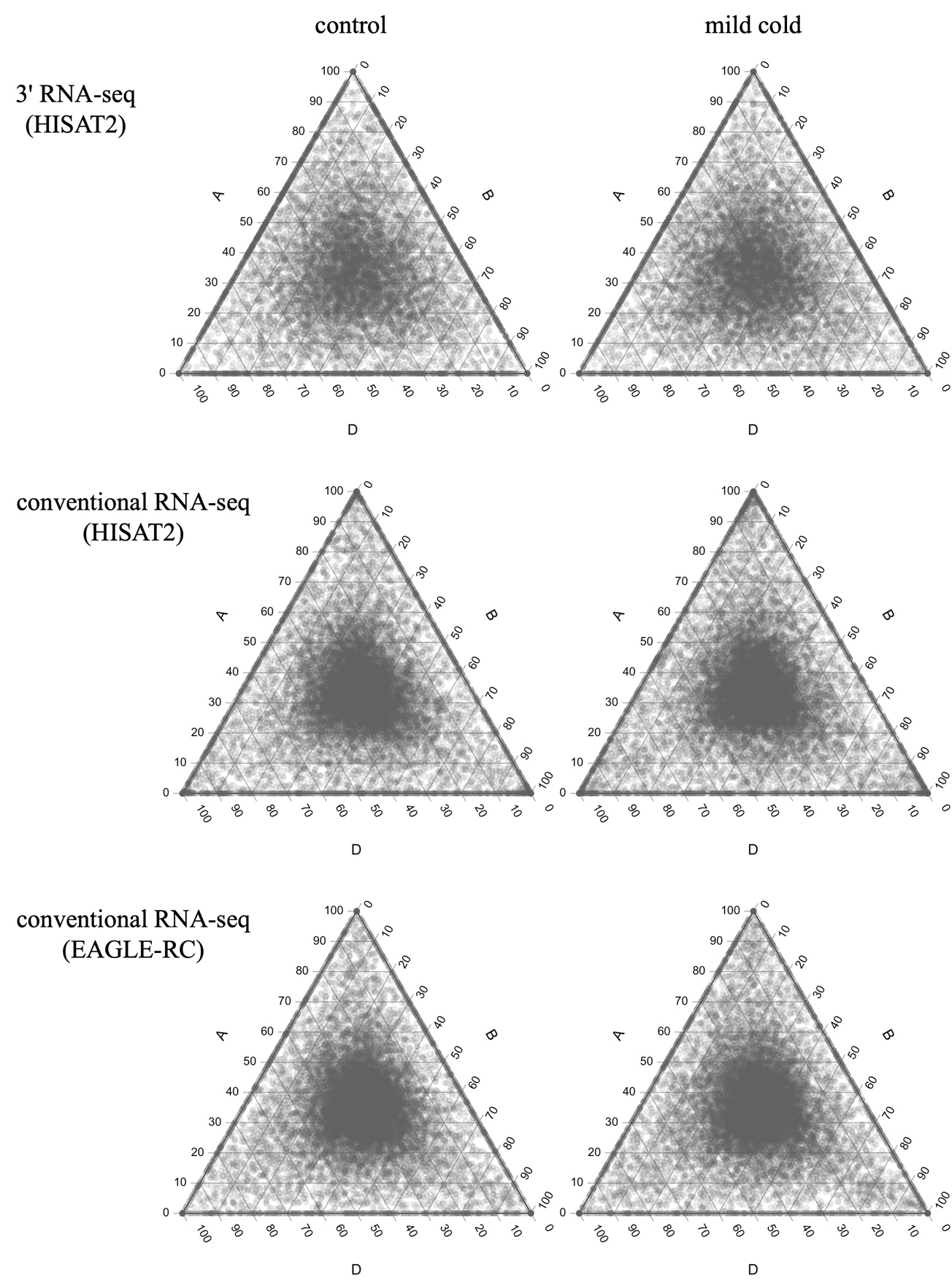

Supplement: lqad067_Supplemental_Files [file lqad067_supplemental_files.zip › README.docx]
